# *Allium ursinum*: botanical, phytochemical and pharmacological overview

**DOI:** 10.1007/s11101-013-9334-0

**Published:** 2013-12-25

**Authors:** Danuta Sobolewska, Irma Podolak, Justyna Makowska-Wąs

**Affiliations:** Department of Pharmacognosy, Jagiellonian University, Medical College, 9 Medyczna Street, Kraków, Poland

**Keywords:** *Allium ursinum*, Biological activity, Ramson, Steroidal saponins, Sulfur compounds

## Abstract

Ramson—*Allium ursinum* L. is a medicinal and dietary plant species with a long tradition of use. This mini-review summarizes the current knowledge on the phytochemistry and pharmacological properties of this valuable plant, with special emphasis on antimicrobial, cytotoxic, antioxidant, and cardio-protective effects.

## Introduction

*Allium ursinum* L. has been used for centuries in traditional medicine. However, studies on its composition and pharmacological activity are fairly recent and scarce. The aim of the present review was to summarize the most important aspects related to *A. ursinum* and provide an outline of phytochemical and pharmacological properties of this relatively poorly known plant species of the *Allium* genus.

The species name “*ursinum*” is of Latin origin, being derived from “*ursus*” (bear), and is related to folk tales, according to which bears after awakening from winter hibernation consume this plant to remove toxins from the body and to regain strength (Rejewski 1996). Another etymological hypothesis refers to the “Ursa major”, a constellation in the northern hemisphere, perhaps because *A. ursinum* was, to ancient Greeks especially, one of the most northerly distributed *Allium* species (Böhling 2008). Contemporary systematics places this plant in the family *Amaryllidaceae* (previously in *Alliaceae*), subgenus *Amerallium* Traub, section *Arctoprasum* Kirschl. (Friesen et al. 2006; Chase et al. 2009; Govaerts 2011). Several synonyms are recognized: *Allium nemorale* Salisb., *A. latifolium* Gilib., and *Ophioscorodon ursinum* (L.) Wallr. Thanks to its wide-spread distribution and popularity as edible and medicinal plant, most modern European languages have common names for *A. ursinum* which are used interchangeably. These are: Ramson or Bear’s garlic (English); Bärlauch (German); Ail des ours, Ail sauvage (French); Лyк мeдвeжий, Чecнoк мeдвeжий or Чepeмшa (Russian); Ramslök (Swedish); Daslook (Dutch); Czosnek niedźwiedzi (Polish).

Also, the name “wild garlic” is very often used in literature with respect to *A. ursinum*, though it can be sometimes ambiguous, since it also refers to other species, like *A*. *vineale* or *A*. *canadense*, as well as to plants from the genus *Tulbaghia* (Defelice 2003; Maine Rare Plant List 2013; Lyantagaye 2011).

## Description

As far as morphological features are concerned ramson is a typical representative of the *Allium* genus. The plant is a bulbiferous, vernal geophyte. Its bulb is narrow, elongated, about 1.5–6 cm long, surrounded by the layers of clear skin with only a few fibers at the base. Sometimes daughter bulbs are formed, what is important for vegetative reproduction. Contractile roots start to develop approximately from the age of three (Eggert 1992; Ernst 1979; Szafer et al. 1988; Oborny et al. 2011; Macků and Krejča 1989; Činčura et al. 1990). When the soil is soft enough to enable the roots to dwell deeper and deeper, after 10 years they can reach down the level even 27 cm lower (Ellenberg 1988). Ramson grows up to the height of 50 cm. The aerial parts of the plant consist of a triquetrous, erect, flower stem, solid in cross-section. Atop a stalk, there is a semispherical umbel-like inflorescence, which comprises of 3–30 starry, snowy-white flowers (according to Błażewicz-Woźniak: 13.4–24.0 on average). They are surrounded by 2–3 spathal bracts until anthesis. Flower parts are in 6’s sets (Eggert 1992; Ernst 1979; Berger 1960; Szafer et al. 1988; Rejewski 1996; Macků and Krejča 1989; Činčura et al. 1990; Błażewicz-Woźniak et al. 2011). Ramson’s blooming usually starts in April and ends in the first half of May.

The plant develops 2–3 leaves, which are shorter than stem, smooth, flat, elliptic-lanceolate with a distinct, well-developed blade, sharpened at the apex, and gradually narrowed into petiole at the base. The width of ramson leaves is 20–64 mm (Szafer et al. 1988; Błażewicz-Woźniak and Michowska 2011). A comparative study on a collection of *A. ursinum* specimens from different ecotypes in Poland (Dukla, Roztocze, Bieszczady) showed that, they differed significantly in the width of leaf blades, the length of leaf stalks and flowering stems, the number of flowers in inflorescences (Błażewicz-Woźniak and Michowska 2011).

*Allium ursinum* regenerates mainly by seeds; vegetative regeneration is of minor importance. The seeds are black, subglobose, 2–3 mm wide, gathered in trichotomic capsules (Hermy et al. 1999; Sendl 1995). Mean weight per seed is 5.4 ± 0.7 mg. They are shed in June and July, however shedding time may be delayed by weather conditions, e.g. a cold spring and summer, or north-facing aspect (Ernst 1979). Most of the seeds fall onto the ground directly beneath the capsules, but they seem to be too heavy to be moved by the wind at a ground level (Oborny et al. 2011; Ernst 1979). So, for a long-distance transport to potential growing sites the participation of animals or running water is needed (Eggert 1992). Most of the seeds remain dormant for 1 or 2 years, however, some germinate in the course of upcoming winter or spring, usually from November to March (according to Ernst it takes place from January to April) (Eggert 1992; Ernst 1979). A dense carpet of *A. ursinum* can produce a large number of seeds annually, even 10,000 seeds/m^2^ as was recorded in Gottingen Forest (Germany) (Ernst 1979). In Litovelské Pomoraví (Czech Republic) floodplain forest, the mean seed production was estimated as 2,692 seeds per m^2^ (max 5,612 seeds/m^2^) (Rychnovská and Bednář 1998).

## Geographical distribution and habitat requirements

*Allium ursinum* is a perennial herbaceous species, of wide-spread distribution both in Europe and Asia. Although, not growing at high altitudes (beyond 1.900 m) and in the far North (beyond ca 64°N), it can be found on natural stands from the Mediterranean region to Scandinavia (Oborny et al. 2011). It is also native to Asia Minor, the Caucasus, and Siberia, up to the Kamchatka Peninsula (Rola 2012; Madaus 1938; Oborny et al. 2011; Djurdjevic et al. 2004).

Two subspecies of *A. ursinum* are recognized: *A. ursinum* ssp. *ursinum* and *A. ursinum* ssp. *ucrainicum*. The subdivision is based on the smoothness of pedicel surface (Karpaviciene 2006). The pedicels of the ssp. *ursinum* are scabrid, with numerous papillae, and rough, while the ssp. *ucrainicum* has smooth pedicels without papillae (Rola 2012; Farkas et al. 2012). The former is distributed in western and central Europe, whereas the latter in eastern and southeastern part of the continent (Rola 2012; Oborny et al. 2011). The distribution areas of the two subspecies can overlap what results in the existence of transitional forms. In Poland, both subspecies along with the intermediate forms, occurring at the border of the distribution ranges, were recorded (Rola 2012). In West Caucasus and Germany ssp. *ucrainicum* is sometimes transplanted into gardens (Hanelt and Büttner 2001). Also, cultivation trials have been made in former Czechoslovakia.

The habitat preferences are equal for both subspecies (Rola 2012). Ramson—flourishes best in light and medium, nutrient-rich, damp, but well-drained soils, in full shade and semi-shade localities (Szafer and Zarzycki 1972; Činčura et al. 1990; Oborny et al. 2011). Although the plant prefers high air humidity, it can be also found on shallow, and fairly dry, in summer months especially, calcareous soil (Eggert 1992). However, both waterlogging and drought are considered as restricting factors for ramson (Kovacs 2007). Another factor limiting its distribution is the aluminium concentration in the soil water (Andersson 1993). Under experimental conditions aluminium (at the concentration of 20 μM) severely restricted ramson root elongation. Also, the growth of new roots was poor. Ramson often forms dense populations covering large areas in horn-beam-oak and beech forests, it can even become monodominant over big areas in the herb layer in the woods (Szafer and Zarzycki 1972; Činčura et al. 1990; Oborny et al. 2011; Morschhauser et al. 2009). *A. ursinum* is considered as one of the species, the patchy distribution of which is a characteristic feature of the herb layer in the *Hordelymo*-*Fagetum* forest community. Under the dense carpets of these species, competition for space and light is likely to occur (Leuschner and Lendzion 2009). Furthermore, *A. ursinum* is a strong inter-specific competitor, which affects the growth of other herbaceous plants via soil, where phenolic phytotoxins (produced by ramson) are accumulated. It competes with other plant species also through volatile compounds (Djurdjevic et al. 2004). Experimental data from the seeds germination and seedlings growth tests have shown that aqueous extract and volatile compounds of ramson bulbs inhibited other plants (lettuce, amaranth and wheat) growth stronger than extract from the leaves (Djurdjevic et al. 2004).

The period of active growth of *A. ursinum* lasts 3.5–4 months starting in early spring, between late February and early March, before the full development of tree leaves (e.g. in the northern Vienna Woods—approximately 60 days before) (Jandl et al. 1997). This provides enough of light in the first stage of the plant growth and helps to avoid competition for light with the canopy (Shmanova and Krichfalushii 1995; Oborny et al. 2011). In late spring however, the canopy cover protects the plant from direct sunlight and helps to keep the appropriate humidity of the topsoil air. Rapid development of ramson stands is associated with high assimilation rate, and accumulation of nutrients stored in the bulbs (Jandl et al. 1997). The above-ground parts abruptly wither as summer arrives. The mortality rate of *A. ursinum* during the first 2 years of life is estimated at around 21 % (Bierzychudek 1982). In the following year it increases as the plant develops contractile roots which are more subjected to attack by insects and nematodes (Ernst 1979). It is estimated that only about 1–10 % of ramson seedlings reach the reproductive age (Bierzychudek 1982). The estimated average life span and age of the first sexual reproduction for *A. ursinum* are 8–10 and 4–5 years respectively.

## Chemical constituents

*Allium ursinum* has a distinct garlic-like scent associated with the presence of sulfur-containing compounds which are the most characteristic constituents in *Allium* plants.

### Sulfur compounds

These are undoubtedly the most important ramson’s constituents, both in terms of chemotaxonomic value and pharmacological activity. Their qualitative and quantitative profile is subject to great variation, the predominant reason for which is their lability. Of the various sulfur compounds present in this, as well as other *Allium* species, glutamyl peptides and sulfoxides are considered as primary. Usually, these plants contain a high concentration of *S*-alk(en)yl-l-cysteine-sulfoxides—odorless, non-volatile sulfur secondary metabolites, which, following subsequent hydrolysis, give rise to many volatile compounds, including thiosulphinates and (poly)sulfides responsible for specific *Allium* flavor and odor (Boscher et al. 1995; Yoo and Pike 1998).

**Fig. 1:**
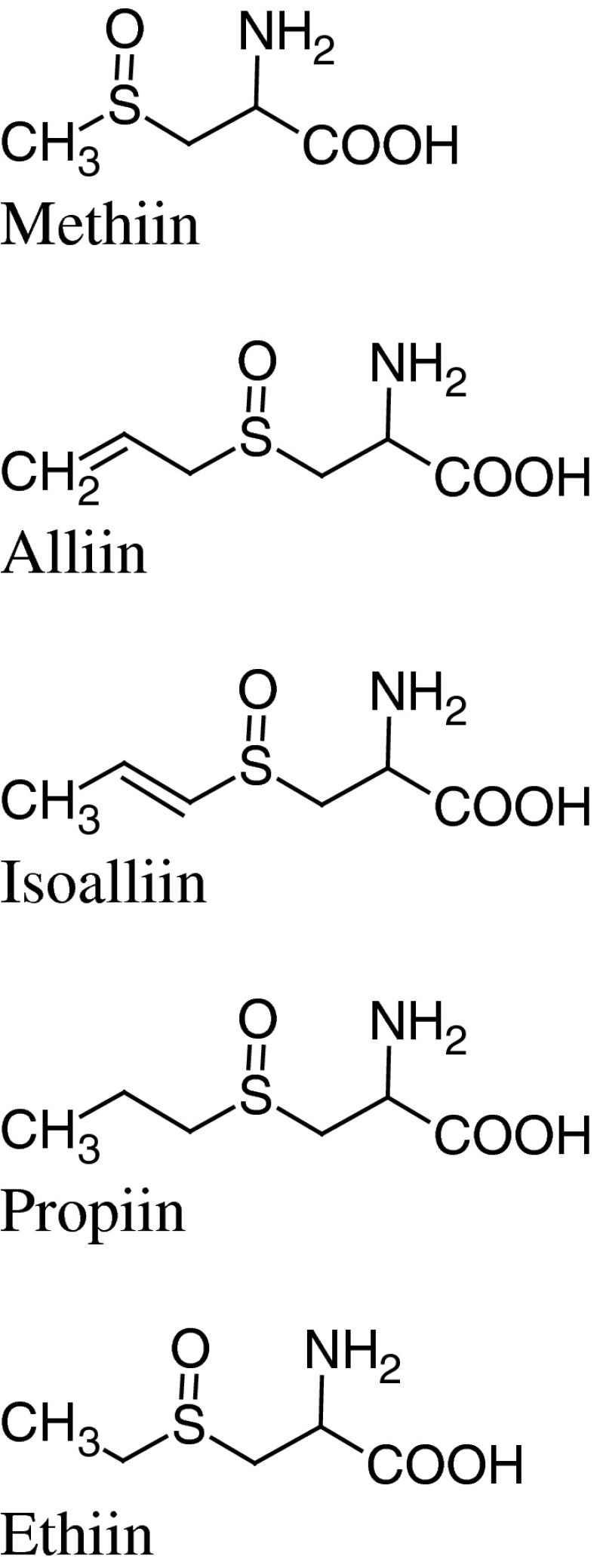
*Allium ursinum* cysteine-sulfoxides

Ramson belongs to a methiin/alliin-type *Allium* species, which means it contains mainly a mixture of (+)-*S*-methyl-l-cysteine-sulfoxide (methiin) and (+)-*S*-allyl-l-cysteine-sulfoxide = (+)-*S*-2-propenyl-l-cysteine-sulfoxide (alliin) (Schmitt et al. 2002; Kubec et al. 2000). However, isoalliin—(+)-*S*-(1-propenyl)-l-cysteine-sulfoxide and propiin—(+)-*S*-propyl-l-cysteine-sulfoxide are present as well (Fig. 1) (Schmitt et al. 2002). Also, ethiin (*S*-ethyl-cysteine-sulfoxide) was reported in the sample of fresh leaves collected in April in Czech Republic (Kubec et al. 2000).

The quantitative profile of cysteine-sulfoxides depends on the plant organ and time of harvest. Their total content in the leaves collected in April, expressed as mg/100 g of fresh weight, was 101.9 (in this: methiin—60.0, ethiin—0.4, propiin—1.2, alliin—40.3, isoalliin—traces) (Kubec et al. 2000). The content of total cysteine sulfoxides in the bulbs harvested in late summer, calculated as alliin, was 0.26 % (amount was related to the fresh weight) (Keusgen et al. 2003). In water extracts from cloves and leaves containing hydrolytic enzyme inhibitor, the alliin content ranges were: for cloves—0.65–1.10 and for leaves 0.20–0.72; while methiin: 0.60–1.40 and 0.30–0.95, respectively (Sendl 1995). The relative proportions of cysteine sulfoxides in ramson are presented in Table 1 (Fritsch and Keusgen 2006).

**Table 1:** The relative proportions of cysteine sulfoxides in leaves and bulbs of ramson (adapted from Fritsch and Keusgen 2006)

Total content (average) (%)	Relative methiin (%)	Relative alliin (%)	Relative isoalliin (%)	Relative propiin (%)	Origin of material
0.245	39	24	37	0	Urft in der Eifel
0.164	35	28	37	0	

The analysis of the changes in the total cysteine sulfoxides content in different parts of *A. ursinum* collected in Germany throughout the vegetation period (focused on the months from March to June) showed that the highest amounts in leaves, storage leaves and bulbs (0.42, 0.26, 0.38 % respectively) were reached in March and April, that is before flowering time (Schmitt et al. 2002, 2005). Samples of fruits and leaf stalks collected in June contained 0.25 and 0.15 % of cysteine sulfoxides (fw) respectively. Furthermore, the relative quantitative profile of the investigated sulfoxides (alliin, methiin, isoalliin and propiin) differed during the vegetation period. In March the bulb contained almost the same amounts of alliin and methiin. In the following weeks alliin became the main component (73 ± 18 %), while methiin content decreased to 15 ± 9 % in mid-May (Schmitt et al. 2005). Then, the rise of methiin levels was observed. The relative content of propiin was always below 5 %, while of isoalliin ~10 %.

As was mentioned above, cysteine sulfoxides are subject to hydrolytic cleavage leading to a formation of a number of characteristic volatile secondary products. This is performed by specific enzymes, named C,S-lyases, which catalyse the cleavage of the Cβ–Sγ bond of the sulfoxides. In the intact cell, cysteine sulfoxides are localized in the cytoplasm, while the hydrolytic enzyme is found in vacuoles. Cellular compartmentalization damage results in its release, and subsequent hydrolysis of sulfoxides (Boscher et al. 1995; Sendl 1995). This reaction occurs upon tissue damage, e.g. when the organ is crushed, minced, or otherwise processed, or in case of the pathogens attack (Ankri and Mirelman 1999).

C,S-lyase isolated from ramson has a molecular mass of 150,000 Da and consists of three subunits (Landshuter et al. 1994). The optimal pH and temperature for its activity were established at 6.0 and 35 °C, respectively. After 30 min. of incubation at pH 3.0 ramson’s C,S-lyase lost its activity by 90 %. Nevertheless, its enzymatic properties are maintained at low temperatures, what was observed even after ramson cloves were freezed at −20 °C. It seems that such feature of the C,S-lyase system provides all year long protection for the underground parts of the plant after disruption of the parenchyma. In contrast to similar enzymes isolated from *A. sativum*, *A. cepa* or *A. porrum*, ramson’s C,S-lyase is not a glycoprotein. This was revealed as no interaction with Concanavalin-A-Sepharose, and no staining by periodic acid Schiff-reagent, were observed. The enzyme was however sensitive to low hydroxylamine concentrations. The ramson’s C,S-lyase catalyses *S*-alk(en)yl-cysteine-sulfoxides hydrolysis, with alliin as the most preferred substrate (Landshuter et al. 1994). Its comparison with common garlic’s alliinase showed that even though it is less specific to alliin, it still shows higher relative activity towards other cysteine sulfoxides, especially methiin and isoalliin (Schmitt et al. 2005). The primary products formed as a result of C,S-lyases action are thiosulfinates, pyruvic acid, and ammonia.

Thiosulfinates are regarded as primarily responsible for odor and flavor of freshly prepared ramson macerates, and of the products obtained by both the extraction and an unorthodox kind of distillation conducted at room temperature (Block et al. 1992). The results showed that room temperature steam distillation provides half the amount of thiosulfinates obtained by direct extraction (Block et al. 1992).

**Fig. 2:**
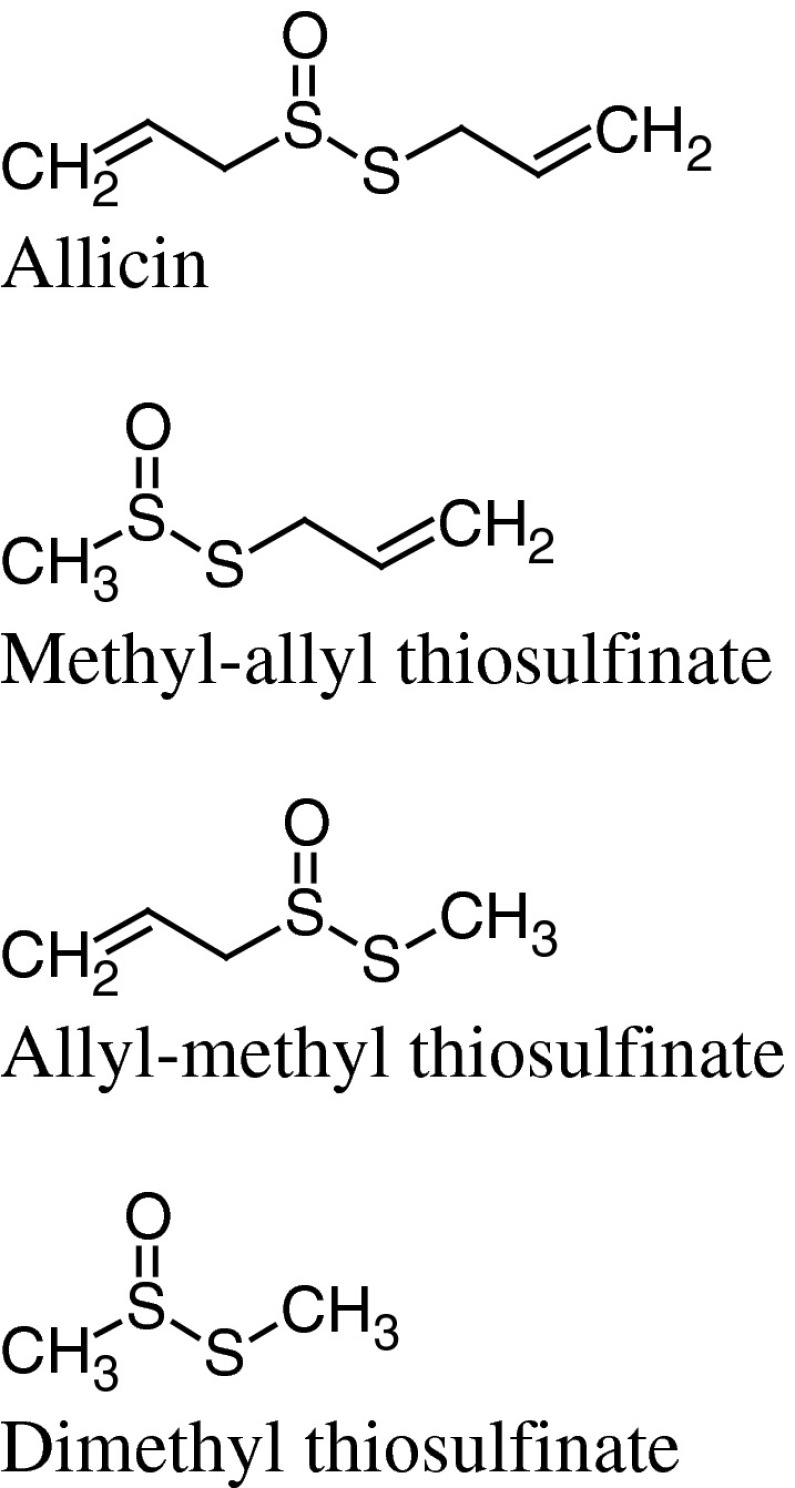
Major thiosulfinates found in ramson extracts

The major thiosulfinates found in ramson extracts are allicin (diallyl thiosulfinate = di-2-propenyl thiosulfinate), and methyl-allyl- or dimethyl thiosulfinates (Fig. 2) (Sendl and Wagner 1991). According to Sendl they constitute 75–90 % of all the compounds formed immediately after hydrolysis of cysteine sulfoxides (Sendl 1995). Data from quantitative HPLC determination of thiosulfinates in chloroform extracts from leaves and bulbs, refered to dry weight, is presented in Table 2 (Sendl and Wagner 1991). In a ramson freeze-dried powder total thiosulfinates concentrations expressed as molar percentage of total was 21 (based on the weight of powder) (Block et al. 1992).

**Table 2:** Data from quantitative HPLC determination of thiosulfinates in chloroform extracts from leaves and bulbs of *Allium ursinum* (adapted from Sendl and Wagner 1991)

Plant material	Allicin (%)	MATS (%)	DMTS (%)
*A*. *ursinum* bulbs	0.53	0.70	0.27
*A*. *ursinum* leaves	0.13	0.26	0.13

*MATS* allyl-methyl and/or methyl-allyl thiosulfinate, *DMTS* dimethyl thiosulfinate

Thiosulfinates are unstable, reactive compounds, that easily decompose to (poly)sulfides, dithiins, ajoenes, and other volatile and non volatile compounds. This takes place on storage, during processing, e.g. in the presence of organic solvents, and also when heat-treated. Allicin is very unstable even at room temperature. Studies by Bagiu et al. (2010) showed that after 20 h at 20 °C it decomposed completely resulting in di-2-propenyl disulfide, di-2-propenyl trisulfide, di-2-propenyl sulfide, and sulfur dioxide.

**Fig. 3:**
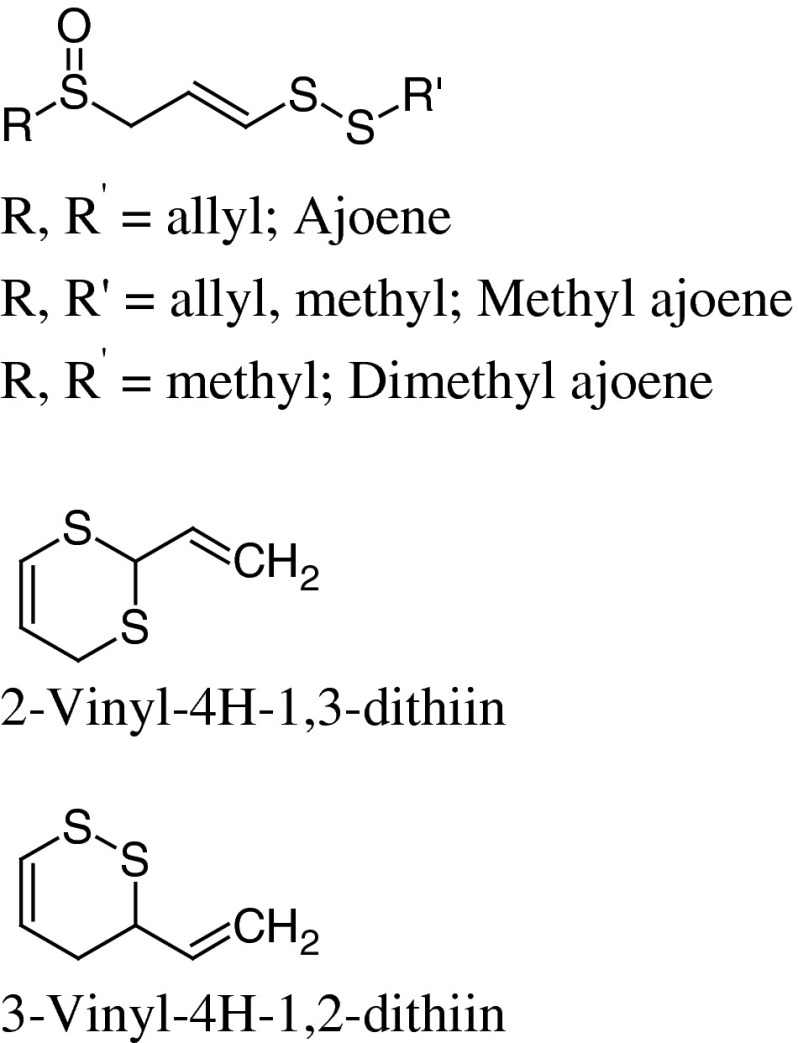
Ajoenes and dithiins present in *A. ursinum* extracts

Vinyldithiins are cyclic compounds which are another group of degradation products of allicin. They are formed as reaction products in solvents less polar than 2-propanol, e.g. hexane (Sendl 1995). It was also observed that pure allicin due to its thermolabile nature degraded to vinyldithiins during GLC analysis. In the hexane extract from the bulbs of *A. ursinum* Wagner and Sendl (1990) identified 2-vinyl-4H-1,3-dithiin and 3-vinyl-4H-1,2-dithiin (Fig. 3). Vinyldithiins (3,4-dihydro-3-vinyl-1,2-dithiin and 2-vinyl-4H-1,3-dithiin) were identified also in the essential oil isolated from the leaves and flowers of ramson samples collected in Bulgaria (Ivanova et al. 2009).

Another group of thiosulfinates degradation products, namely ajoene, methyl- and dimethyl ajoenes, were identified in acetone–chloroform extracts from ramson bulbs (Fig. 3). Comparative analysis of *A. sativum* and *A. ursinum* extracts showed that ajoene dominated in garlic, while its methyl- and dimethyl homologues were the main components in ramson extract (Wagner and Sendl 1990). It should be mentioned that ajoenes and vinyldithiins were found as well in oil-macerated garlic (Benkeblia and Lanzotti 2007).

Apart from the above-mentioned sulfur-containing sulfoxides degradation products, various sulfur compounds have also been detected as constituents of ramson’s essential oil. This was obtained (0.007 %) for the first time already in 1887 by Semmler, who identified alkyl sulfides, alkyl polysulfides, and trace amounts of mercaptan (Sendl 1995).

The amount of oil varies depending on soil condition, geographical location, and part used. For example, isolation of essential oil from fresh and air-dried leaves and flowers of ramson collected in Bosnia yielded 0.011 % (w/w) and 0.024 % (w/w) respectively (Copra-Janicijevic et al. 2008). The results of qualitative analyses of essential oils from ramson collected from different sites in Europe showed significant differences in their composition (Table 3). Furthermore, different time of harvest and analytical method applied influenced the profile of investigated oils.

**Table 3:** Composition of essential oils isolated from *Allium ursinum* leaves collected at different sites in Europe (Ivanova et al. 2009; Godevac et al. 2008; Schmitt et al. 2005; Błażewicz-Woźniak and Michowska 2011)

Compounds	Origin of plant material					
	*1	*2	*3	*4	*5	*6
Sulfide, methyl 2-propenyl			+			
Sulfide, di-2-propenyl			+			
Propylene sulfide	+					
Disulfide, methyl 2-propenyl	+	+	+	+	+	+
Disulfide, methyl propyl		+	+	+	+	+
Disulfide, methyl 1-propenyl, (*E*)-		+	+	+	+	+
Disulfide, methyl 1-propenyl, (*Z*)-			+			
Disulfide, dimethyl	+		+	+	+	+
Disulfide, dipropyl			+			
Hex-3-en-1-ol, (*E*)-		+	+			
Disulfide, di-2-propenyl	+	+	+	+	+	+
Disulfide, 2-propenyl propyl	+	+	+			
Allyl (*E*)-1-propenyl disulfide		+	+			
Disulfide, 1-propenyl propyl, *cis*			+			
Disulfide, 1-propenyl propyl, *trans*			+			
Nonanal		+		+	+	
Trisulfide, methyl 2-propenyl	+	+		+	+	+
Trisulfide, methyl propyl		+	+			
Trisulfide, dimethyl	+	+	+	+	+	+
Trisulfide, methyl 1-propenyl, (*Z*)-		+				
Trisulfide, methyl 1-propenyl, (*E*)-		+				
Tetrasulfide, dimethyl	+	+		+	+	+
Trisulfide, dipropyl	+		+			
Trisulfide, di-2-propenyl	+	+		+	+	+
Trisulfide, propyl 2-propenyl		+				
Trisulfide, 1-propenyl 2-propenyl, (*Z*)-		+				
Trisulfide, 1-propenyl 2-propenyl, (*E*)-		+				
Tetrasulfide, methyl 2-propenyl		+		+	+	+
Tetrasulfide,di-2-propenyl	+	+		+	+	+
1-Propene, 3,3′-thiobis-(CAS#592-88-1)					+	+
Propylthiol			+			
Dimethylthiophene	+		+			
Pentanal, 2-methyl			+			
2-Hexenal	+		+			
Cumene					+	+
Benzene, 1-ethyl-4-methyl					+	
1,2,3-Trimethylbenzene				tr.	+	+
5-Hepten-2-one, 6-methyl					+	
*n*-Octanal				tr.	+	+
Cyclohexanone, 2,2,6-trimethyl				+	+	
1-Propene, 1-(methylthio)-, (*Z*)-				+	+	+
1-Propene, 1-(methylthio)-, (*E*)-				+	+	tr.
Decenal				+	+	tr.
1-Cyclohexene-1-acetaldehyde, 2,2,6-trimethyl				tr.	+	
2H-1-benzopyran 3,4,4a,5,6,8a-hexahydro-2,5,5,8a-tetramethyl, (2α,4a α α,8aα)-				+	+	+
2,6,10,10-Tetramethyl-1-oxa-spiro[4,5]dec-6-ene				+	+	
2-Undecanone, 6,10-dimethyl				tr.	+	
(*E*)-β-caryophyllene				+	+	
Geranyl acetone				+	+	+
Alloaromadendrene				+	+	
(*E*)-β-ionone				+	+	+
2-Tridecanone				+	+	+
1,6,10-Dodecatrien-3-ol, 3,7,11-trimethyl					+	
2-Hexadecanol					+	
Spathulenol				+	+	+
Caryophyllene oxide				+	+	+
1-Tetradecanal	+			+	+	+
Pentadecanal				+	+	+
2-Pentadecanone, 6,10,14-trimethyl				+	+	+
5,9,13-Pentadecatrien-2-one, 6,10,14-trimethyl, (*E*,*E*)-				+	+	+
Phytol	+			+	+	+
*n*-Hexadecanoic acid				+	+	+
Phytol acetate				+	+	+
2-Vinyl-1,3-dithiane	+					
3,4-Dihydro-3-vinyl-1,2-dithiin	+					
2-Vinyl-4H-1,3-dithiin	+					
2-Hexenol	+					
1-Hexadecanal	+					
1-Octadecenol	+					
1-Octadecen	+					
Heneicosane	+					
Tricosane	+					
Tetracosane	+					
Pentacosane	+					
Hexacosane	+					
Heptacosane	+					
Nonacosane	+					
Phytol isomer	+					

*tr.* Traces

*1 Leaves harvested in the vicinity of Ihtiman, Bulgaria; the oil obtained by hydrodistillation method; GC/MS analysis (Ivanova et al. 2009)

*2 Leaves collected in the vicinity of Belgrade, Serbia; hydrodistillation; GC/MS analysis (Godevac et al. 2008)

*3 The samples collected in the area of Quedlinburg, Germany; the investigation by SPME-GC (Schmitt et al. 2005)

*4 The plant material harvested in Dukla, Poland

*5 Roztocze region, Poland

*6 Bieszczady, Poland; isolation of the oil by steam distillation; GC/MS analysis (Błażewicz-Woźniak and Michowska 2011)

From among over 20 components identified in the volatile oil of *A. ursinum* collected in Serbia, the most abundant fraction was disulfides (54.7 %), followed by trisulfides (37.0 %), tetrasulfides (4.7 %), and the non-sulfur components (1.0 %) (Godevac et al. 2008). The composition of essential oils of three ecotypes of *A. ursinum* collected in Poland also differed significantly in the dominant components (Błażewicz-Woźniak and Michowska 2011). The Roztocze ecotype contained methyl-2-propenyl disulfide (16.05 % on average), 6,10,14-trimethyl-2-pentadecanone (13.55 %), nonanal (11.93 %), and dimethyl trisulfide (12.07 %) as main components. The Dukla ecotype oil was composed mainly of phytol (17.03 %) and *n*-hexadecanoic acid (16.57 %), while in the oil of the Bieszczady ecotype phytol acetate (16.40 %) and (*E*)-β-ionone (13.33 %) dominated (Błażewicz-Woźniak and Michowska 2011). The results of SPME-GC analysis of ramson oil, from the leaves collected in the area of Quedlinburg (Germany) showed that diallyl disulfide was the major component, amounting to ~50 % (Schmitt et al. 2005). Allyl-methyl disulfide, allyl-methyl sulfide, diallyl sulfide, and (*E*)-allyl-1-propenyl disulfide were abundant, as well. However, according to the authors, the SPME method did not supply information on some other volatile substances, such as 2-hexenal or hex-3-en-1-ol, which were detected by an SDE-GC method. GC/MS analysis of samples from fresh flowers collected in the vicinity of Ihtiman (Bulgaria), showed that the main components of the volatile fraction were (*E*)-methyl-2-propenyl disulfide, methyl-2-propenyl trisulfide, dimethyl trisulfide, 3,4-dihydro-3-vinyl-1,2-dithiin and 2-vinyl-4H-1,3-dithiin (Ivanova et al. 2009). Schmitt et al. (2005) detected the decrease of the relative yield of oil (sum of volatile substances) during the vegetation period, and observed that this was in agreement with the decreasing amounts of cysteine sulfoxides. An especially significant decrease in allyl methyl disulfide and dimethyl disulfide levels was seen, while the relative content of (*E*)-allyl-1-propenyl disulfide increased.

Apart from studies on the composition of steam-distilled essential oil from ramson, a very interesting aspect is investigation of the atmospheric emission rate of organic sulfur compounds. Such studies were conducted in a Viennese suburban forested park in which *A. ursinum* was grown as ground cover (Puxbaum and König 1997). Sulfur emission rates (μg S) per gram of dry weight and per unit of ground area were 1 μg/g × h and 60 μg/m^2^ × h, respectively. The authors claimed this was the highest rate ever reported for such substances emitted from a terrestrial plant.

### Phenolics

Apart from sulfur-containing substances *A. ursinum* has been also reported to be a good source of phenolic compounds. It should be mentioned, however that the extraction method may substantially alter the level of active compounds isolated. Total polyphenol content, expressed as gallic acid equivalents (GAE), in the leaf extract obtained by a 12-day maceration with 70 % ethanol at room temperature (20 °C) was higher in comparison with the one prepared by the ultrasound-assisted extraction: 27.9 g GAE/100 g dry basis versus ~10 g GAE/100 g (Gîtin et al. 2012). Total free phenolics content in the leaves was determined as 3.24 mg/g, while in the bulbs 2.30 mg/g. The amount of bound forms was about the same in the leaves and in the bulbs (1.10 and 1.00 respectively) (Djurdjevic et al. 2004). Gross differences were also noted in studies on gallic acid levels. Its qualitative and quantitative analysis in hydroalcoholic extracts from *A. ursinum* leaves showed that: 96 % methanol extract had a gallic acid content 0.0576 mg/ml, 80 % methanol extract—0.0165 mg/ml; while 96 % ethanol extract—0.0076 mg/ml (Condrat et al. 2010).

The studies on the content of phenolic acids in fresh leaves and bulbs of ramson collected in an experimental forest situated in West Serbia exhibited some differences between free and bound compounds in these plant parts (Djurdjevic et al. 2004). The amounts of free phenolic acids in leaves and bulbs were 119.75 and 180.91 μg/g, respectively, while of bound forms—135.30 and 248.97 μg/g, respectively. The leaves contained free forms of ferulic and vanillic acids, and bound forms of *p*-coumaric, ferulic and vanillic acids. In the bulbs free ferulic, *p*-hydroxybenzoic and vanillic acids, and bound forms of *p*-coumaric and ferulic acids were detected.

The flavonoid content (expressed as mg quercetin equivalent—QE) determined in fresh leaves collected in March, from the Bacau city forests (Romania), using ultrasound-assisted extraction was ~7.3 mg QE/kg fresh plant; while using conventional maceration—2.7 mg QE/kg (Gîtin et al. 2012). Also, the total content of flavonoids in the different parts of *A. ursinum* collected in June in the forest area near Wrocław (Poland) differed significantly: seeds—73.14 mg/100 g of dry mass, stalks—206.07 mg/100 g, green leaves—1,856.31 mg/100 g, yellow leaves 2,362.96 mg/100 g (Oszmiański et al. 2013).

As far as qualitative profile is concerned, ramson is abundant predominantly in kaempferol derivatives. The ethanol extract from the leaves collected near Laceno Lake (Italy) yielded: 3-*O*-β-neohesperidoside-7-*O*-[2-*O*-(*trans*-*p*-coumaroyl)]-β-d-glucopyranoside (1), 3-*O*-β-neohesperidoside-7-*O*-[2-*O*-(*trans*-*p*-feruloyl)]-β-d-glucopyranoside (2), 3-*O*-β-neohesperidoside-7-*O*-[2-*O*-(*trans*-*p*-coumaroyl)-β-d-glucopyranosyl]-β-d-glucopyranoside (3), 3-*O*-β-neohesperidoside-7-*O*-β-d-glucopyranoside (4), 3-*O*-β-neohesperidoside (5) (Carotenuto et al. 1996). From the *n*-butanol fraction of the dry leaves of ramson collected in Denmark seven flavonoid glycosides were isolated. Three of them have been previously reported by Carotenuto et al. (compounds 1, 4, 5). The remaining were also identified as kaempferol derivatives: 3-*O*-β-d-glucopyranoside, 3-*O*-β-d-glucopyranosyl-7-*O*-β-d-glucopyranoside, 3-*O*-α-l-rhamnopyranosyl-(1 → 2)-[3-acetyl]-β-d-glucopyranoside and 3-*O*-α-l-rhamnopyranosyl-(1 → 2)-[6-acetyl]-β-d-glucopyranoside (Wu et al. 2009). Compounds 4, 5, kaempferol 3-*O*-β-d-glucopyranoside and kaempferol 3-*O*-β-d-glucopyranosyl-7-*O*-β-d-glucopyranoside were isolated from the *n*-butanol extract from fresh flowers (Ivanova et al. 2009). The analysis of flavonoid content in acidified methanol extracts from green and yellow leaves, stalks and seeds collected in June in the forest area near Wrocław (Poland) led to the isolation of 21 compounds, all kaempferol derivatives (Oszmiański et al. 2013).

### Steroidal glycosides

**Fig. 4:**
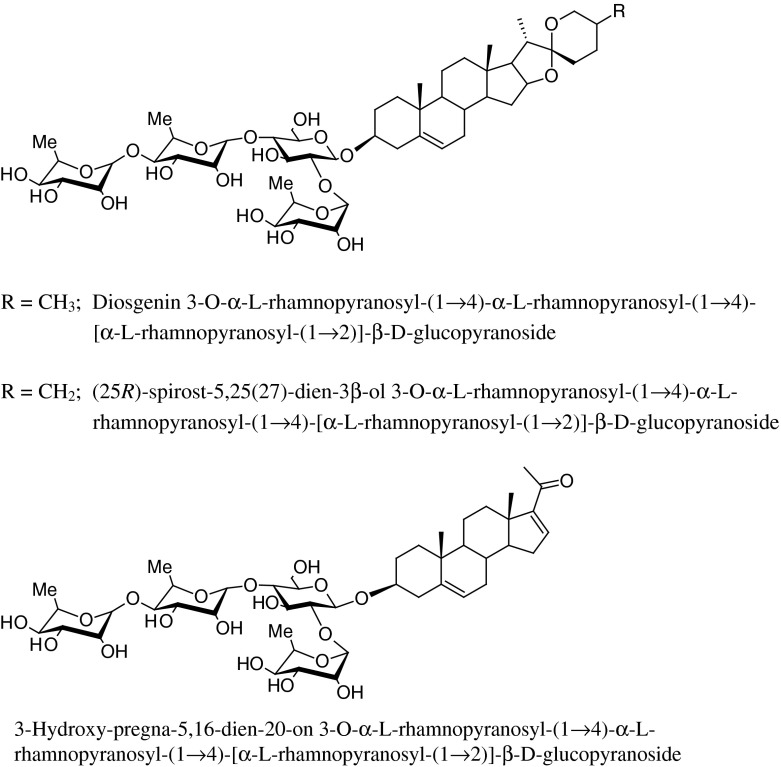
Steroidal glycosides isolated from the bulbs of *A. ursinum*

Similarly to organosulfur compounds, steroidal saponins are also commonly found in the *Allium* genus. The following were reported in the bulbs of *A. ursinum*: diosgenin 3-*O*-α-l-rhamnopyranosyl-(1 → 4)-α-l-rhamnopyranosyl-(1 → 4)-[α-l-rhamnopyranosyl-(1 → 2)]-β-d-glucopyranoside and (25*R*)-spirost-5,25(27)-dien-3β-ol 3-*O*-α-l-rhamnopyranosyl-(1 → 4)-α-l-rhamnopyranosyl-(1 → 4)-[α-l-rhamnopyranosyl-(1 → 2)]-β-d-glucopyranoside (Fig. 4) (Sobolewska et al. 2006). A pregnane glycoside: 3-hydroxy-pregna-5,16-dien-20-on 3-*O*-α-l-rhamnopyranosyl-(1 → 4)-α-l-rhamnopyranosyl-(1 → 4)-[α-l-rhamnopyranosyl-(1 → 2)]-β-d-glucopyranoside has been identified as well (Fig. 4) (Sobolewska et al. 2006).

Diosgenin content in *A. ursinum* depended on the part of the plant and the time of harvest (Sobolewska et al. 2009). Methanol extract prepared from fresh bulbs collected in April, prior to flowering, yielded the highest content of diosgenin (0,137 %). In the extract made from leaves collected at the same time, the amount of diosgenin was 10 times lower, while in the one from leaves collected in March and June it was not detectable. Low diosgenin content in ramson does not make this plant species a valuable source for the isolation of this sapogenin.

In an ethanol extract from fresh leaves β-sitosterol 3-*O*-β-d-glucopyranoside was found (Sabha et al. 2012).

### Other

Other interesting constituents identified in *A. ursinum* include lectins, which were isolated from bulbs, roots and leaves collected in April (Smeets et al. 1997). Root compounds were identical to those found in bulbs: AUAI, which is a heterodimeric lectin composed of polypeptides of 12.5 and 11.5 kDa, and AUAII a homodimeric lectin composed of polypeptides of 12 kDa. Both lectins are mannose-specific, and show a good reaction with synthetic (1 → 3) and (1 → 6) mannans. The ramson leaf lectin (AUAL) differs from the bulb lectins, and also from the leaf-specific lectins identified in other *Allium* species. It is a dimer composed of 12 kDa subunits.

The bulbs are also rich in polysaccharides. According to Hegnauer, they may contain as much as 30–90 % of mostly fructans (Hegnauer 1963). In the bulbs harvested between May and August the content of fructan U, consisting of fructose residues only, was estimated as 75–90 % (Meier and Reid 1982). Unfortunately, no modern structure elucidation studies on this compound have been performed to date. The studies on reserve carbohydrates in *A. ursinum* from the Sheffield flora (United Kingdom) resulted in determination of the maximum fructan concentration in the bulbs harvested in summer, as 139 mg/g fresh weight (Hendry 1987).

**Fig. 5:**
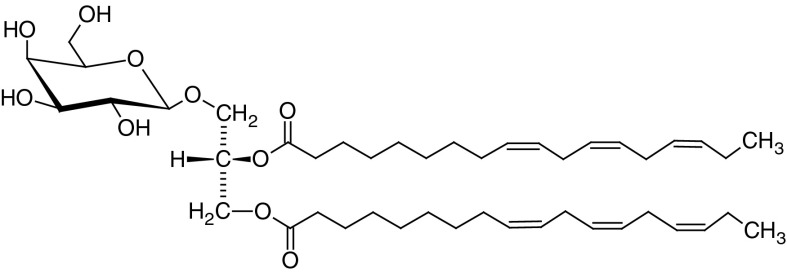
2-Di-*O*-α-linolenoyl-3-*O*-β-d-galactopyranosyl-sn-glycerol (DLGG) found in an ethanol extract from fresh leaves of *A. ursinum*

A number of fatty acids were reported in the hexane extract from the bulbs. These were palmitic, linoleic, oleic, palmitoleic, stearic, α-linolenic, and myristic acid (Wiater et al. 1998). Moreover, water extracts yielded fairly rare, but pharmacologically valuable γ-glutamylpeptides, and many amino acids, such as: asparagine, glutamine, aspartic acid, glutamic acid, arginine, alanine, glycine, threonine (Wagner and Sendl 1990). In an ethanol extract from fresh leaves 2-di-*O*-α-linolenoyl-3-*O*-β-d-galactopyranosyl-sn-glycerol (DLGG) (Fig. 5), was identified (Sabha et al. 2012).

Ramson leaves seem to be relatively abundant in pigments, as compared to other *Allium* plants, the content of which amounts to: 2.87 ± 0.03 mg/g of chlorophyll a, 1.35 ± 0.01 mg/g of chlorophyll b, and as much as 9.99 ± 0.01 mg/g of carotenoids (Štajner and Szöllosi Varga 2003). Comparative analysis of some macro- and microelements in *A. ursinum* and *A. sativum* showed that ramson contained higher levels of magnesium (7,000 mg/kg), manganese (1,600 mg/kg) and iron (230/mg/kg) than garlic (6,114, 952, 14 mg/kg, respectively). *A. ursinum* is a rich source of adenosine (120 mg/kg) (Nagori et al. 2010).

*Allium ursinum* L. ssp. *ucrainicum* floral nectar volume and concentration were investigated in three different habitats in the Mecsek hills (South Transdanubia, Hungary) (Farkas et al. 2012). The study revealed that ramson produces low to medium volumes (ranged 0.1–3.8 μl) of highly concentrated nectar (25–50 % sugar concentration). Freely sun-exposed flowers produced lower quantity of nectar than covered flowers at a given time. The higher volume of nectar with higher sugar content was observed in populations living in optimal life conditions for *A. ursinum* (the sessile oak-hornbeam association). The plants living in the silver lime-flowering ash rock forest, where the lack of sufficient nutrients was observed, produced lower quantities of nectar.

## Uses

Ramson has been used for centuries to promote general health, and as the old English proverb says:

Eat leeks in Lide [March] and ramsons in May

And all the year after the physicians may play.

There is good evidence for the use of ramson by Mesolithic people. Charred bulbs of *A. ursinum* were identified—in the late Mesolithic settlement at Halsskov in Denmark (Kubiak-Martens 2002). It was hypothesized that ramson was one of the plants that contributed to the hunter-gatherer diet. *A. ursinum* was known to the early Celts and to the ancient Romans. The Greek physician Dioscorides mentioned four kinds of onion, among them *A. ursinum* and also attributed a detoxifying effect to the plant (Meyer et al. 1999; Richter 1999). Ramson was well known also in the Middle Ages; it belongs to the group of plants often found at medieval West Slavic archeological sites (Celka 2011). King Charles the Great, also known as Charlemagne, included *A. ursinum* in his *Capitulare de Villis imperialibis*, where he formally cataloged plants, mostly those possessing medicinal properties, and documented how the gardens should be planned and cared for (Clickner 2011). Hieronymus Bock provided drawings of the plant in his *Kreutterbuch*, Lonicerus judged wild garlic to be superior to regular garlic (Richter 1999; Błażewicz-Woźniak et al. 2011; Strzelecka and Kowalski 2000; Madaus 1938).

All parts of the plant are edible. For medical purposes leaves/herb—*Allii ursini folium*/*herba*, collected in April and May, and bulbs—*Allii ursini bulbus*, collected in September and October, are used. Ramson is usually collected from the wild. However, in Poland this species, which belongs to the group of 11 alliaceous plants growing wild there, has been partially protected since 2004 and is listed in the “Red list of plants and fungi in Poland”, what made it impossible to be wild-harvested (Szafer et al. 1988; Zarzycki and Mirek 2006).

In European traditional medicine ramson has been generally recommended as digestive stimulant, antimicrobial agent, removing toxins from the body, and to prevent cardiovascular diseases (Treben 1992; Macků and Krejča 1989; Leporatti and Ivancheva 2003). It was often applied as a remedy in respiratory problems, such as common cold with fever or bronchitis. *A. ursinum* has been effective when used externally to support wound healing, in chronic skin disorders, and in acne.

In recent years there has been a growing interest in its use as a dietary supplement and food. There are some records that in the nineteenth century Switzerland butter made from milk of cows fed on ramson were used. Such milk tasted slightly of garlic. Apparently in Eberbach in Germany there is a festival called Bärlauchtage—Bear’s Garlic Days, which is devoted to this plant. Today, it is a common practice to use ramson in cuisine. Fresh leaves can be eaten raw or cooked, and as a kind of pesto. They are often added to soups, gnocchi, risotto, ravioli, and as a spice to flavor hard cheeses or spreads based on cottage cheeses. Leaves and flowers can be used as a garnish to salads, while ramson’s bulbs can be used like common garlic.

*Allium ursinum* is also a component of dietary supplements available on the European market. For example, it is one of constituents found in the recipes used therapeutically in the University Hospital of Bucharest (Romania) (Epure et al. 2011). Such products as Api Ursomax and Memo Ursomax are recommended as detoxifying and antiatherogenic medicines. The former is additionally advertised as a mineralizing agent, while Memo Ursomax is claimed to be a memory stimulant.

## Pharmacological studies

Modern pharmacological studies have confirmed many of the above mentioned traditional indications of ramson. For example, a great number of in vitro and in vivo experiments showed that *A. ursinum* is a plant with a high potential for the prevention and treatment of cardiovascular system diseases. Different extracts obtained from the fresh leaves of *A. ursinum* were tested in vitro on human platelet aggregation. The results showed a significant inhibitory activity of the ethanol extract on ADP-induced aggregation. The mechanism of action was similar to that of a reference drug Clopidogrel (Hiyasat et al. 2009). It was suggested, that the active compounds exerting antiaggregatory effect are 1,2-di-*O*-α-linolenoyl-3-*O*-β-d-galactopyranosyl-sn-glycerol (DLGG) (Fig. 5) and β-sitosterol 3-*O*-β-d-glucopyranoside (Sabha et al. 2012). DLGG has previously been identified in a number of medicinal and food plants, and has been shown to possess anti-inflmmatory activity (Larsen and Christensen 2007).

**Fig. 6:**
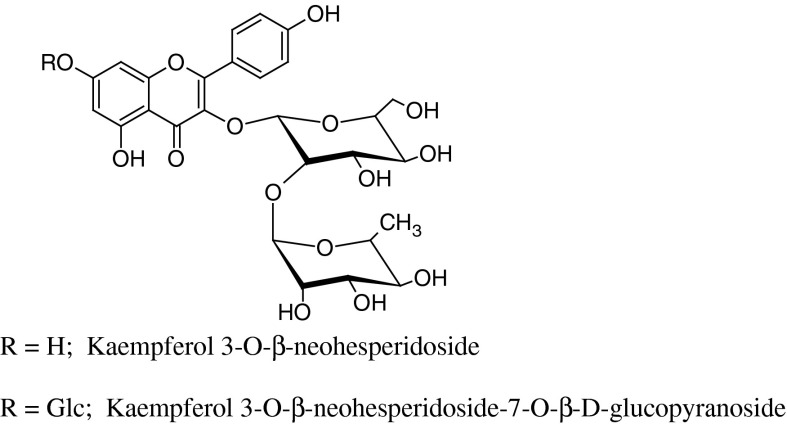
Flavonoids exerting in vitro inhibitory activity on platelet aggregation induced by collagen

Moreover, two of the flavonoids present in ramson leaves: kaempferol 3-*O*-β-neohesperidoside-7-*O*-β-d-glucopyranoside and 3-*O*-β-neohesperidoside (Fig. 6), showed in vitro inhibitory activity on platelet aggregation induced by collagen (Carotenuto et al. 1996). As other kaempferol glycosides were inactive, it was concluded that the presence of *p*-coumaroyl group in the molecule and the increase in the number of monosaccharides in the sugar residue deplete the antiplatelet potential of these compounds.

Ramson’s administration affects also the activity of ACE. In vitro tests on the water extract from the leaves (at the concentration of 0.3 mg/ml), showed higher inhibition of this enzyme activity as compared to garlic leaves extract (58 vs. 30 %) (Sendl et al. 1992a). This probably resulted from the differences in glutamyl peptides contents. The in vitro study on the effect of the ramson essential oil on the artificial liposome membrane model demonstrated that the fluidity of the membrane close to the surface was statistically non-significantly changed, while in deeper layers the fluidity increased (Godevac et al. 2008). The authors postulated that further studies should be continued to estimate the role of *A. ursinum* volatile oil in the regulation of membrane functions in hypertension.

In vivo experiments on rats fed for 8 weeks standard diet with 2 % of pulverized *A. ursinum* leaves showed significantly lower plasma ACE activity in the ramson group as compared to control (Rietz et al. 1993). The studies performed on Spontaneously Hypertensive Rats (Okamoto strain) that were fed with diet containing 1 % w/w ramson (Pfannenschmidt, Inc. of Hamburg) showed that after 45 days it reduced final mean systolic blood pressure when compared to control (173 ± 0.7 vs. 189 ± 1.2 mm Hg respectively) (Preuss et al. 2001). Diet enrichment with ramson was more effective than with garlic at the same concentration (the final SBP—175 ± 1.2 mm Hg). *A. ursinum* decreased elevated circulating insulin concentration and total cholesterol level, however HDL tended to increase. Similarly, when both garlics were consumed at lower concentrations—0.1 % (w/w)—systolic blood pressure readings at 10, 18, and 26 days were significantly lower in rats consuming ramson compared to the animals consuming common garlic. Authors concluded that these effects may be associated with high concentration of glutamyl peptides, adenosine or phenolic compounds in ramson. They suggested that consuming *A. ursinum* may result in a greater therapeutic benefit when compared to *A. sativum* at a given concentration. Animal studies demonstrated that ramson-containing diet may reduce the size of the ischemic zone and ischemia and reperfusion—induced arrhythmias (Rietz et al. 1993).

Ramson showed in vitro inhibitory activity on cholesterol synthesis. Chloroform and chloroform/acetone extracts from *A. ursinum* bulbs, at concentrations of 166 μg/ml, inhibited cholesterol biosynthesis by 49.3 and 48.9 %, respectively. The results were nearly identical to those obtained for garlic extracts. Of the pure investigated components present in the extracts ajoene, methyl ajoene, 2-vinyl-4H-1,3-dithiin and allicin were the strongest cholesterol synthesis inhibitors, providing at the concentration of 10^−3^ M the inhibition values of 69.5, 72, 58.4, and 52.2 %, respectively (Sendl et al. 1992b). Pharmacological studies have also revealed that chloroform and acetone/chloroform extracts from ramson exerted in vitro inhibitory activity on 5-lipoxygenase and cyclooxygenase, however they were less effective than the corresponding garlic extracts (Sendl et al. 1992a). Thrombocyte aggregation test revealed no differences between *A. ursinum* and *A. sativum* extracts (Sendl et al. 1992a).

As was mentioned above, *A. ursinum* has been valued in traditional medicine as an antimicrobial agent used either internally or externally. There is a substantial number of reports in which the antimicrobial activity of various extracts prepared from different plant parts were tested in vitro against a wide array of bacterial and fungal strains.

Comparative analysis of water and methanol extracts from ramson herb (at the concentration range 0.16–83.7 and 0.06–35.5 mg/ml, respectively) showed that the latter was more active against microbes. It inhibited the growth of the following bacteria: *Staphylococcus aureus*, *Bacillus subtilis*, *Escherichia coli*, *Proteus mirabilis*, *Salmonella enteritidis*, and fungi: *Cladosporium* sp., *Aspergillus niger*, *Rhizopus nigricans*, *Geotrichum candidum*, *Penicillium expansum*, *Candida lipolytica*, *Mycoderma*, *Saccharomycopsis fibuligera* (Synowiec et al. 2010). The average antibacterial MIC value was 35 mg/ml with the exception of *S. aureus* ATTC 25923 strain, in the case of which the MIC was 17.7 mg/ml. The highest antifungal effect was observed against *C. lipolytica* (MIC = 8.9 mg/ml), whereas for other tested strains it was less pronounced (MIC = 17.7 mg/ml), however still much higher in comparison to the water extract (concs. 41.9–83.7 mg/ml). The antibacterial activity of the water extract was seen only against *B. subtilis* ATTC 6633 (at 83.7 mg/ml). A water extract (at pH 7.0, adjusted with 0.1 mol/l K_2_HPO_4_) from *A. ursinum* leaves exhibited antibacterial activity in vitro against *Listeria monocytogenes*, *S. aureus*, *E. coli*, and *Salmonella enterica* subsp. *enterica* (Sapunjieva et al. 2012). The inhibition zones were greater in the case of Gram (+) bacteria.

A comparative analysis of the in vitro germination and growth inhibitory effects of the ethanol extracts from flowers and leaves of *A. ursinum* against *A. niger*, *Botrytis cinerea*, *Botrytis paeoniae*, *Fusarium oxysporum* f.sp. *tulipae*, *Penicilium gladioli* and *Sclerotina sclerotiorum* showed that the flower extract possessed the highest antifungal activity (MIC 100, 60, 70, 140, 90, and 60 μg/ml, respectively). The authors claimed that the antifungal effects of the extracts could be positively correlated with allicin content: 1.946 mg allicin/ml flower extract versus 0.028 mg allicin/ml leaf extract (Parvu et al. 2011). Pure allicin at concentrations 1.57–6.25 μg/ml showed inhibitory activity against *Candida*, *Cryptococcus*, *Trichophyton*, *Epidermophyton*, and *Microsporum* strains (Ankri and Mirelman 1999).

Antimicrobial activity of the bulb juice of *A. ursinum* was correlated with storage temperature and pH levels. Its activity against selected bacteria and fungi decreased on storage at the temperature above 4 °C and with an increase in the pH value (Tynecka et al. 1993).

The antimicrobial activity of different extracts (acetone, chloroform, ethyl acetate, *n*-butanol and water) from fresh flowers and leaves of Bulgarian ramson was studied. Acetone extracts from both parts and chloroform extract from the leaves were active against *S. aureus* (MIC 625 μg/ml), while none of the extracts inhibited the growth of *E. coli*. The chloroform extract from the leaves showed inhibitory effect on *Candida albicans* (MIC 312 μg/ml), as well (Ivanova et al. 2009). The fresh water extract from the bulbs inhibited the growth of different *Candida* strains, with MIC ranging from 1 mg/ml to 4 mg/ml depending on the particular yeast strain. The adhesion of *Candida* ssp. isolates to catheters (silicone-elastomer—coated latex urinary Foley catheter and PCV Thorax catheter) was not prevented by the extract even at the maximal concentration of 4.0 mg/ml (Chudzik et al. 2010). The extracts prepared from fresh *A. ursinum* leaves collected in Romania during blossoming phase inhibited the growth of *Candida* ssp. (*C. albicans*, *C. famata*, *C. glabrata*, *C. krusei*) at concentrations ranging from 0.5 to 4.0 mg/ml (Bagiu et al. 2010).

The broad spectrum of antimicrobial activity of *Allium* plants is generally associated with sulfur-containing compounds, however our own studies have shown that other constituents may as well contribute to that effect, to some extent. The inhibitory activity of a mixture of diosgenin tetrasaccharide and (25*R*)-spirost-5,25(27)-dien-3β-ol tetrasaccharide isolated from the bulbs against *Candida glabrata* and *C. parapsilosis* was determined, with MIC values of 200 and 250 μg/ml, respectively (Sobolewska et al. 2003). Both compounds however, were ineffective against *Pseudomonas aeruginosa* and *A. niger* at concentrations up to 400 μg/ml, by the disc diffusion method. With regard to antifungal properties against *Trichophyton mentagrophytes* and *Microsporum canis* the saponin mixture was active at the concentration 400 μg/ml (Sobolewska et al. 2006).

There were also some studies which evaluated the potential of ramson against parasites. For example, the juice from the bulbs was effective against free living nematode *Rhabditis* sp., larvae of *Nippostrongylus brasiliensis*, and hindered the development of *Ascaris suum* eggs (Chybowski 1997).

Isolated ramson’s lectins were assessed for potential inhibitory effect against HIV-1- and HIV-2-induced cytopathicity in MT4 cells (Smeets et al. 1997). The EC_50_ values (the concentration required to protect MT4 cells against cytopathicity of HIV by 50 %) of bulbs and leaf lectins were about 3 and 5 μg/ml for HIV-1 and HIV-2, respectively. The specific agglutination activity (the lowest concentration which still yields a visible agglutination of a 1 % suspension of erythrocytes) of AUAL, AUAI and AUAII was the same (being 1.2 μg/ml). *A. ursinum* lectins were more potent agglutinins than the *A. sativum* bulb lectins ASAI and ASAII (specific activities being 6 and 100 μg/ml, respectively), but less active than the garlic leaf lectin (0.2 μg/ml).

The occurrence in various parts of the plant of constituents with well-known antioxidant properties, such as flavonoids or carotenoids, urged investigations that would confirm ramson’s antioxidative potential. As was shown, extracts from different parts exhibited high free radicals scavenging activity. The antioxidant effect of ramson leaves may be associated not only with the presence of phenolic compounds but also with high activity of antioxidant enzymes, like catalase and peroxidase (11.48 ± 2.90 U/mg protein and 8.85 ± 0.19 U/mg protein, respectively), whereas in the bulbs, with superoxide dismutase (31.43 ± 6.96 U/mg protein) (Štajner et al. 2008; Štajner and Szöllosi Varga 2003).

Also, the volatile oil of ramson has been tested, however it demonstrated poor antioxidant activity against DPPH^+^ and ABTS^+^ in comparison to BHT (butylated hydroxytoluene) and Trolox. On the other hand, in the beta-carotene-linoleic bleaching test the oil showed an effect comparable to that of BHT (Godevac et al. 2008). Based on these results, the authors concluded that the antioxidant effect depends on the method used, and also on which free radical generator or oxidant is involved (Godevac et al. 2008). It seems therefore mandatory to employ different analytical methods that would varying oxidation initiators and targets.

Nevertheless, some isolated ramson volatile oil constituents have revealed promising antioxidant properties. Diallyl disulfide increased the intracellular content of reduced glutathione in rat red blood cells, while diallyl sulfide enhanced the activity of antioxidative enzymes, and activated Nrf2 protein, what resulted in suppression of inflammatory cytokines (Wu et al. 2001; Kalayarasan et al. 2009).

Other pharmacological activities which were reported for *A. ursinum* include in vitro cytotoxicity. Nine different extracts (chloroform, methanol, and water) prepared by hot extraction of fresh leaves, flowers, and flower stems were analysed in vitro against murine cancer cell lines melanoma B16 and sarcoma XC (Trypan Blue Exclusion Test of Cell Viability) (Sobolewska et al. 2012). The methanol extracts from the aerial parts and the aqueous extracts from leaves and flowers were inactive or only slightly active over the entire concentration range (10–200 μg/ml) against both cell lines, while the aqueous extracts from flower stems showed no activity towards melanoma B16 cells. The chloroform extract from flower stems showed the most promising cytotoxic activity: at the concentration of 60 μg/ml of this extract 100 % of melanoma B16 cells were killed after 24 h, while at the concentration of 20 μg/ml—after 48 h. In both cell lines colchicine had an ED_50_ value lower than 2 μg/ml (0.5 ± 0.003—melanoma B16; 1.5 ± 0.005—sarcoma XC) after 24 h (Sobolewska et al. 2012). Moreover, cytotoxic activity of a mixture of diosgenin tetrasaccharide and (25*R*)-spirost-5,25(27)-dien-3β-ol tetrasaccharide on melanoma B16, sarcoma XC and human fibroblasts HSF was evaluated as well. The saponin mixture was found active against murine melanoma B16 and sarcoma XC. It exhibited 100 % effect at 2 μg/ml on both strains. It showed no activity towards human fibroblasts HSF at concentrations below 3 μg/ml (Sobolewska et al. 2006).

Diallyl disulfide (a component of ramson volatile oil) inhibited the proliferation of various human cancer cell lines, including breast, lung, colon cancers, lymphomas and neuroblastoma. The mechanism of action involved cell cycle arrest or apoptosis. Also, diallyl trisulfide induced apoptosis in human prostate cancer cell lines PC-3 and DU-145 (Lai et al. 2012).

## Adverse reactions

Generally, *A. ursinum* is recognized as safe. However, there is some evidence of hemolytic anemia due to oxidative damage to erythrocytes following the consumption of other Alliums by domestic and farm animals (Munday et al. 2003). It seems that diallyl tri- and tetrasulfides, which are highly toxic to erythrocytes, may be largely responsible for this effect, and these compounds are present in ramson volatile oil, as well. Even though there were no reports of hemolysis associated with *Allium* plants consumption in humans, certain individuals whose erythrocytes are unusually vulnerable to oxidative damage, should consume garlic with caution. Case reports of allergic reactions to some garlic constituents have been also described (Borelli et al. 2007), and such compounds as diallyl disulfide, allylpropyl sulfide and allicin were identified as allergens. All are present in various *A. ursinum* preparations as well. The study on garlic-allergic patients in Taiwan revealed that garlic C,S-lyase (alliinase) was a major *Allium sativum* allergen (Kao et al. 2004). As this enzyme showed cross-reactivity with C,S-lyases from other species, the authors concluded that this molecule is a common allergen in *Allium* plants. The potential of *A. ursinum* to enhance existing anticoagulant therapy should be taken into consideration, as well.

Even though the garlic-like odor of ramson should enable its unambiguous identification, it should be noted that there were some cases of fatal poisoning by ingestion of toxic plants, the leaves of which, due to a similar shape, were mistakenly wild-harvested as ramson. These were, in particular, autumn crocus (meadow saffron, *Colchicum autumnale*), the lily-of-the-valley (*Convallaria majalis*), and white hellebore (*Veratrum album*) (Colombo et al. 2010; Sundov et al. 2005; Gilotta and Brvar 2010; Klintschar et al. 1999).

## Conclusions

Despite centuries of use of ramson as a substitute for garlic (*A. sativum*), pharmacological studies on *A. ursinum* bulbs and leaves have begun fairly recently, that is about 20 years ago. Thus, data referring to *A. sativum*, which is a species much valued for its therapeutic potential are much more abundant, even though the conclusions related to its clinical efficacy are often inconsistent. A broad spectrum of biological activities recorded for ramson extracts and the presence of chemical compounds with high therapeutic potential, makes this plant species a possible candidate for future development as a medicinal product. Undoubtedly, some problems that may appear are associated with producing a uniform plant material as ramson composition is very sensitive to changes in growth conditions what could hinder large scale production and standardization. Nevertheless, it is worth noting, that definitely in recent years as been recognized as a valuable spice plant.
